# Outcomes of endovascular aortic arch repair with an off-the-shelf modular inner branched stent-graft: an IDEAL 2a prospective multicentre trial

**DOI:** 10.1093/bjs/znaf279

**Published:** 2026-01-09

**Authors:** Wei Guo, Dan Rong, Hongkun Zhang, Leiyang Zhang, Hui Zhuang, Hua Peng, Xuejun Wu, Kunmei Gong, Wei Wang, Zhen Li, Weiguo Fu, Xiaoming Zhang, Mingjin Guo, Guangqi Chang, Xiangchen Dai, Jian Zuo, Yingqiang Guo, Bing Chen, Lei Zhang, Taoran Zhang, Hongpeng Zhang

**Affiliations:** Department of Vascular and Endovascular Surgery, Chinese PLA General Hospital, Beijing, China; School of Medicine, Nankai University School of Medicine, Tianjin, China; Department of Vascular and Endovascular Surgery, Chinese PLA General Hospital, Beijing, China; Department of Vascular Surgery, The First Affiliated Hospital of Zhejiang University School of Medicine, Hangzhou, China; Department of Thoracic and Cardiovascular Surgery, Nanjing First Hospital, Nanjing, China; Department of Vascular Surgery, Xiamen Cardiovascular Hospital Xiamen University, Xiamen, China; Department of Vascular Surgery, Xiamen Cardiovascular Hospital Xiamen University, Xiamen, China; Department of Vascular Surgery, Shandong Provincial Hospital, Jinan, China; Department of Vascular Surgery, The First People’s Hospital of Yunnan Province, Kunming, China; Department of Vascular Surgery, Xiangya Hospital of Central South University, Changsha, China; Department of Endovascular Surgery, The First Affiliated Hospital of Zhengzhou University, Zhengzhou, China; Department of Vascular Surgery, Fudan University Affiliated Zhongshan Hospital, Shanghai, China; Department of Vascular Surgery, Peking University People’s Hospital, Beijing, China; Department of Vascular Surgery, The Affiliated Hospital of Qingdao University, Qingdao, China; Department of Vascular Surgery, The First Affiliated Hospital of Sun Yat-sen University, Guangzhou, China; Department of Vascular Surgery, Tianjin Medical University General Hospital, Tianjin, China; Department of Cardiovascular Surgery, The First Affiliated Hospital of Air Force Medical University, Xi’an, China; Department of Cardiovascular Surgery, West China Hospital, Chengdu, China; Department of Vascular Surgery, The Second Affiliated Hospital of Zhejiang University School of Medicine, Hangzhou, China; Department of Vascular Surgery, The First Hospital of Hebei Medical University, Shijiangzhuang, China; School of Medicine, Nankai University School of Medicine, Tianjin, China; Department of Vascular and Endovascular Surgery, Chinese PLA General Hospital, Beijing, China

## Abstract

**Background:**

Aortic arch pathologies are complex to treat. Alternatives include open surgery, hybrid surgery (endovascular aortic stent-grafting and open surgical debranching procedures) and total endovascular solutions with branched stent-grafts. Branched stent-grafts are the mainstream approach for endovascular repair, but they are primarily available only as dedicated custom-made devices. The aim of this study was to evaluate the safety and effectiveness of a non-customized modular aortic arch stent-graft.

**Method:**

This trial was led by the Chinese PLA General Hospital and 16 additional aortic centres in China. All included patients were treated with a non-customized modular inner branched stent-graft (Endonom Medtech, Hangzhou, China). The study endpoints were 30-day death and stroke, technical success, clinical success, early and late complications, reintervention, and death during follow-up. Follow-up via clinical examination and CT angiography scan were scheduled post surgery at 1, 6, and 12 months, and annually thereafter.

**Results:**

From June 2021 to December 2024, a total of 88 patients were enrolled in this study. Technical success rate was 100%. The mean follow-up was 28.6 ± 11.7 months. The overall 30-day mortality rate was 3%, and the 30-day stroke rate was 9%. Overall survival was 91% ± 3%, 86% ± 4%, and 81% ± 4% at 12, 24, and 36 months respectively. A total of 10 patients developed endoleaks, none of which required reintervention.

**Conclusion:**

Modular branched stent-graft repair for aortic arch disease is feasible and with comparative rates of safety with custom made branched endovascular stent-grafts, hybrid techniques and open surgery. Long-term comparative effectiveness studies are required to establish whether it is superior to alternative interventions.

## Teaser

This study reported a novel modular aortic arch stent graft and included the largest cohort to date, demonstrating significantly lower death compared to open surgery, comparable stroke rates to current arch stent grafts, and lower reintervention rates than current arch stent grafts.

## Introduction

Aortic pathology involving the aortic arch remains a challenge for both open and endovascular repair. Although the gold standard treatment, open surgical repair may not be the best option for high-risk surgical patients because of the need for median sternotomy, core and cerebral cooling, and temporary systemic circulatory arrest. A recent systematic review reported a pooled early mortality rate of 10.4% in 693 patients who received open arch repair^1^. Additionally, age, ejection fraction, previous cardiovascular surgeries, concomitant surgeries, cardiopulmonary bypass time, and cardiac cross-clamp time have been associated with early death after open arch repair^2–4^. Consequently, hybrid approaches without aortic cross-clamping have become alternatives to conventional open repair, but they have not demonstrated improvement in early death or stroke rate compared with conventional open repair^1,5^.

Since the first case of endovascular aortic arch repair was reported in 1999^6^, endovascular repair devices and techniques have been rapidly developed. During this period, the chimney, fenestration, and branched stent-graft techniques have been applied for aortic arch reconstruction. In 2013, the semi-customized Najuta triple-fenestrated stent-graft became the first approved aortic arch device, receiving market approval in Japan^7^. This was followed by the European market approval of the Nexus branched aortic arch stent-graft in 2019^8^. After over 20 years’ development, branched stent-grafts have become the mainstream endovascular solution to reconstruct supra-aortic branches^9–11^. Designed for left subclavian artery (LSA) reconstruction, the Gore Tag Thoracic Branch Endoprosthesis (TBE; W. L. Gore & Associates, Flagstaff, AZ, USA) has also been deployed in innominate artery (IA) reconstruction alongside supra-aortic branch bypass to treat aortic arch pathologies^12^. Other approved options include the custom-made, double-branched NEXUS DUO (Endospan Ltd, Herzliya, Israel) and the triple-branched NEXUS TRE (Endospan, Herzliya, Israel) and Hector (Microport, Shanghai, China) stent-grafts. However, complications, including perioperative stoke, cervical haematoma, and a high rate of reintervention, are still issues of concern. The European Society for Vascular Surgery expert consensus for thoracic aortic pathologies involving the aortic arch recommended endovascular arch repair in patients unfit for open surgery and with a suitable anatomy^13^.

The Zenith (Cook, Bloomington, IN, USA) and Relay (Terumo Aortic, Sunrise, FL, USA) branched arch stent-grafts are the two leading pioneer platforms with customized one-piece main-body design^9,10^. Although a previous study demonstrated that the Zenith branched arch stent-graft can be utilized as an off-the-shelf device in a majority of aortic arch pathologies^14^, both devices are currently available as custom-made devices only. We designed an off-the-shelf modular inner branched stent-graft system (WeFlow-Arch; Endonom Medtech, Hangzhou, China) to reconstruct the aortic arch, for which we have completed an early feasibility study^15^. The aim of this study was to describe the clinical characteristics and treatment details as well as short-term and midterm outcomes of patients with aortic arch pathology who were treated with this novel modular branched stent-graft system.

## Methods

### Study design

A prospective, multicentre study was designed to evaluate the safety and effectiveness of the modular inner branched stent-graft. This study was approved by the National Medical Products Administration and Institutional Review Board of each participating centre, and was conducted in compliance with the Declaration of Helsinki and the Idea, Development, Exploration, Assessment and Long-term follow-up framework (*Table S1*)^16^. The protocol of this study was prospectively registered in the Clinical Trial Registry (NCT04765592) and has been previously published^17^. Market approval for this novel device is being sought in China, and all procedures in this study were performed in strict accordance with the device’s instructions for use (*Fig. S1*). A written consent form was obtained from each patient for employment of the WeFlow-Arch stent-graft system (Endonom Medtech, Hangzhou, China) and anonymous use of relevant data for scientific purposes. This trial was run by the Chinese PLA General Hospital and 16 additional high-volume aortic centres (thoracic endovascular aortic repair > 30 cases per year) in different regions of China.

### Study population

This study applied competitive enrolment. All consecutive patients with aortic arch pathology visiting the participating centres were evaluated by a central multidisciplinary team consisting of a vascular surgeon, cardiovascular surgeon, and anaesthetist at the Chinese PLA General Hospital. When patients were deemed high-risk or declined open surgery, they were further evaluated by investigators to exclude any contraindications for the procedure. Thereafter, a collaborative assessment involving the investigators, imaging specialists, and the stent-graft manufacturer was conducted to determine anatomical suitability. Patients who met all criteria were ultimately enrolled (*Fig. S2*).

The indications for treatment were fusiform or dissection aneurysms with a maximal aortic diameter >50 mm, rapid growth (>10 mm/year), or symptoms regarded as signs of impending rupture; saccular aneurysms of any kind; penetrating aortic ulcers with a maximum diameter >20 mm or a neck >10 mm. The anatomic criteria for endovascular arch repair were: adequate iliofemoral access with a diameter >7 mm with acceptable tortuosity and calcification; an ascending aortic centreline length >50 mm (between the sinotubular junction and ostia of the innominate artery); an ascending aorta diameter ≥24 and ≤44 mm; IA diameter ≤24 and ≥7 mm, with a sealing zone length ≥20 mm; a left common carotid artery (LCCA) or LSA with a diameter ≤24 and ≥7 mm, and a sealing zone length ≥20 mm; an aorta with a shaggy score ≤3 points^18^. The physiological criteria were: no New York Heart Association class IV congestive heart failure; no myocardial infarction or stroke within 6 months; no systemic infection within 3 months; no signs or evidence of connective tissue disease; serum creatinine level <150 μmol/l or estimated glomerular filtration rate ≥45 ml/min/1.73 m^2^; minimum 1-year life expectancy; and no other diseases or abnormalities which would exclude the participant from eligibility.

### Device design

The newly developed modular stent-graft system comprises three key components: a proximal main component (ascending stent-graft), a distal main component (arch stent-graft), and two bridging covered stents (*Fig. S3*). The proximal main component is constructed from a polyethylene terephthalate (PET) graft with an integrated nitinol stent. It features two internal branches for the reconstruction of the IA (10-o’clock position) and LCCA (8-o’clock position). The ascending stent-graft exhibits a diameter range of 26–48 mm, with available lengths of either 50 mm or 60 mm. The internal branch measures 20 mm in length with a diameter of either 12 mm (diameter of the main stent >36 mm) or 10 mm (diameter of the main stent ≤36 mm); the gutter between the two internal branches is sealed with a stitched PET membrane. The ascending stent-graft is preloaded into a 22F or 24F precurved, steerable hydrophilic sheath with a tip capture design. The device is designed to self-orient, ensuring that the inner branches are positioned towards the anterolateral aspect of the ascending aorta.

The bridging covered stents, made of a nitinol stent and an expanded polytetrafluoroethylene membrane, also have a tapered design to adapt to the varying diameters of the supra-aortic artery. The bridging covered stent is designed with single stent cells reinforced by longitudinal metal bars at both proximal and distal ends and a continuous spiral stent cell in the middle, enabling the provision of superior radial support force at the sealing zones with excellent flexibility in the central segment. The proximal diameter of the bridging covered stents was 13 mm for connecting 12 mm inner branches and 11 mm for 10 mm inner branches. The distal diameter ranged from 8 to 26 mm and the length from 60 to 140 mm.

The arch stent-graft is designed with a tapered profile to accommodate the narrower diameter of the distal landing zone in the descending aorta. It is preloaded into a 20F or 22F precurved, steerable hydrophilic sheath, minimizing the need for device manipulation within the aortic arch. The arch stent-graft is available in diameters ranging from 26 to 48 mm and lengths spanning 80–200 mm.

### Procedures

**Fig. 1:**
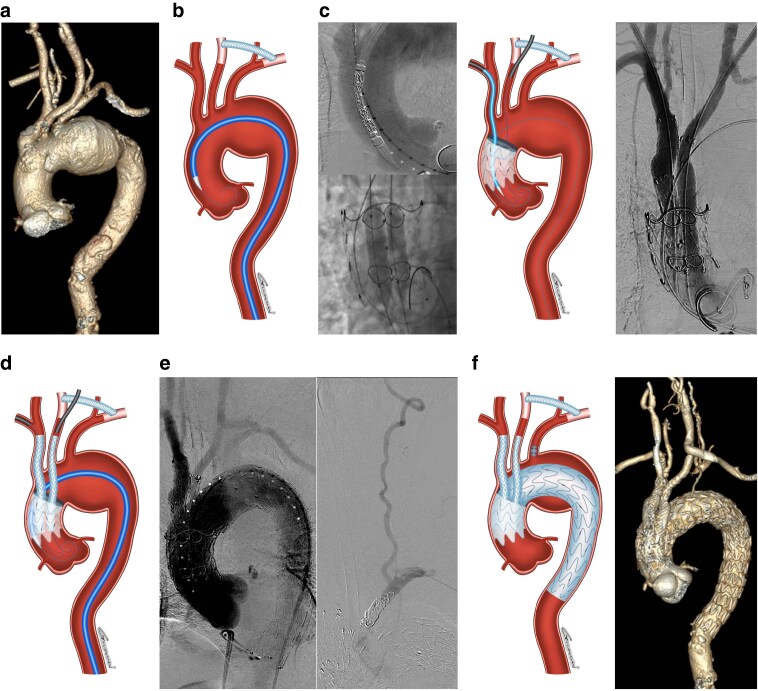
**Case illustration of procedures for implantation of the WeFlow-Arch modular branch stent-graft system (Endonom Medtech, Hangzhou, China)** a Preoperative three-dimensional reconstruction of computed tomography angiogram (CTA) showing an aortic aneurysm involving the aortic arch. b A left common carotid artery (LCCA) to left subclavein artery (LSA) bypass was performed before endovascular procedures. The ascending aortic stent-graft was delivered to the landing zone through a Lunderquist extra-stiff wire (Cook Medical, Bloomington, IN, USA). c After the deployment of ascending aortic stent-graft, the innominate artery (IA) and LCCA inner branches were selectively cannulated, and balloon angioplasty was performed to confirm successful cannulation. Subsequent revascularization of the IA and LCCA was achieved via right brachial artery access and LCCA access respectively. d Following reconstruction of the IA and LCCA, the aortic arch stent-graft was advanced and deployed at the predetermined position to seal the aneurysm. e Proximal LSA coil embolization was then performed via left radial artery access. f Illustration and CTA with three-dimensional reconstruction demonstrated complete exclusion of the aortic arch aneurysm, patency of the bridging covered stents and bypass prosthesis, with no evidence of endoleak.

All procedures were performed under general anaesthesia in a hybrid operating room. An LCCA–LSA bypass was performed prior to the endovascular aortic arch repair in either a staged or simultaneous setting (*Fig. 1a*). If no reconstruction of the LSA was planned, a decision was made following discussion between the operator and the principal investigator after a complete cerebral angiography assessment. Arterial access is needed at four sites for the procedure: percutaneous femoral access for the delivery of the stent-grafts; right brachial, axillary, or carotid artery access to reconstruct the IA; LCCA access to reconstruct the LCCA; and left radial access for LSA embolization.

After systemic heparinization (activated clotting time >250 s), the origin of the IA was marked with a 12F or 14F sheath (COOK or GORE sheaths of appropriate length selected by access route), while the origin of the LCCA was marked with a 12F/30-cm Performer introducer (COOK, Bloomington, IN, USA). A 22F or 24F DRYSEAL introducer sheath (W.L. Gore, Flagstaff, AZ, USA) was placed into the femoral access after two Perclose ProGlide (Abbott Vascular, Redwood City, CA, USA) sutures were prepositioned. From the femoral access, a double-curved extended Lunderquist stiff wire (COOK, Bloomington, IN, USA) was positioned in the aortic sinus or into the left ventricle.

The main component of the stent-graft was flushed with carbon dioxide and subsequently with heparinized saline. After flushing, the ascending stent-graft was advanced into the ascending aorta (*Fig. 1b*). The stent-graft was deployed following systolic blood pressure reduction to the recommended 90 mmHg, achieved through intravenous vasodilators or rapid ventricular pacing. Before tip-capture release, physicians confirmed that the markers of the inner branches were positioned at the 8–10 o’clock in the ascending aorta, ensuring optimal anatomical placement and functional alignment. Following the deployment of the ascending stent-graft, the systemic blood pressure was promptly restored to physiological levels to ensure haemodynamic stability.

The inner branches were selectively catheterized through the right brachial artery access and LCCA access. Successful catheterization was confirmed using dual parallel balloon inflation in the inner branches (*Fig. 1c*). Subsequent reconstruction of the IA and LCCA was achieved using specifically designed bridging covered stents deployed in a retrograde fashion. The arch stent-graft was then delivered through the femoral access and precisely positioned to achieve adequate overlap with the ascending stent-graft, effectively excluding the aortic arch pathologies (*Fig. 1d*). Following this, the origin of the LSA was embolized using either coil systems or vascular occluders (*Fig. 1e*). The procedure was concluded with on table angiography to confirm branch vessel patency and exclude the presence of endoleaks.

### Data collection

Data were collected using a standardized case report form which included medical record data, computed tomography angiography (CTA) imaging data (*Fig. 1f*) and follow-up data. Demographics, co-morbidities, procedural details, and perioperative data were collected before discharge. Follow-up via clinical examination and CTA were scheduled post surgery at 1, 6, and 12 months, and annually thereafter. All data were systematically collected at each participating centre and subsequently consolidated into a unified electronic database. To minimize heterogeneity in the assessment process, CTA images were transmitted to an independent third-party core laboratory in compliance with the standardized Digital Imaging and Communications in Medicine format for centralized analysis and interpretation. Double data entry and cross-validation methods were used to ensure the quality of the data.

The primary endpoint was all-cause death and severe stroke within 12 months after the procedure. Major stroke is defined as a modified Rankin scale score ≥2 at 90 days from stroke onset. The secondary endpoints were technical success, 12-month clinical success (aortic aneurysm diameter growth ≤5 mm), maximum diameter of aortic aneurysm, incidence of endoleaks, stent-graft migration, supra-aortic branch patency, reintervention, and incidence of 30-day major adverse events, including all-cause death, estimated blood loss >1 l, myocardial infarction, ischaemic stroke, paraplegia, acute kidney injury, new-onset dialysis, and respiratory failure.

Participating centres enrolling >10 patients were defined as high-volume centres, whereas those enrolling ≤10 patients were classified as low volume. A comparative analysis between the high-volume and low-volume group was performed. Based on criteria established in a previous study^19^, patients were stratified using an ascending aortic diameter threshold of 38 mm for risk factor analysis.

### Statistical analysis

All study participants successfully completed a minimum follow-up period of 1 year, with no instances of missing data throughout the study duration. Data were presented as mean ± standard deviation or median (range; i.q.r.) for continuous variables and number and percentage for categorical variables. Comparative analyses were performed using the Mann–Whitney test for continuous variables and Fisher’s exact test for categorical variables. The association of ascending aortic diameter with early death and stroke was assessed using multivariable logistic regression models. The inclusion of covariates was guided by both the results of the change-in-estimate screening approach and their clinical relevance. The Kaplan–Meier method was used to estimate the cumulative rate of each endpoint. All data analysis was performed using the statistical software packages R (http://www.R-project.org, The R Foundation) and Free Statistics software version 2.2.

## Results

### Population characteristics

From June 2021 to December 2024, a total of 88 patients from 17 centres were enrolled in the study (*Table S2*), including 76 male (86%) and 12 female (14%) patients with a mean age of 66.8 ± 7.7 years. Co-morbidities comprised typical cardiovascular risk factors, including hypertension (93%), smoking (65%), coronary artery disease (33%), and cerebral artery disease (25%) (*Table 1*). The ASA class was ≥III for 63 patients (72%). The indication for treatment was a degenerative aneurysm in 52 patients (59%), a post-dissection aneurysm in 18 patients (20%), and a penetrating ulcer in 18 patients (20%). Other lesion characteristics, including extent of lesion, arch anatomy, maximum diameter of lesion, and diameter and length of the ascending aorta, are shown in *Table 1*.

**Table 1:** Patient and anatomic characteristics (*n* = 88)

Variable	No. (%) or mean(s.d.)
Age, years	66.8(7.7)
Male gender	76 (86)
BMI, kg/m^2^	24.8(3.0)
Hypertension	82 (93)
Diabetes mellitus	22 (25)
Dyslipidaemia	15 (17)
**Smoking**	
None	31 (35)
Current	35 (40)
Previous	22 (25)
Coronary artery disease	29 (33)
Previous CABG	1 (1)
Previous PTCA or stent	11 (13)
Dysrhythmia	1 (1)
Peripheral artery disease	2 (2)
Previous aortic surgery	4 (5)
Cerebral artery disease	22 (25)
Chronic obstructive pulmonary disease	3(3)
Renal insufficiency	9 (10)
Creatinine, μmol/l	80.8(25.8)
eGFR, ml/min/1.73 m^2^	92.7(28.5)
Ejection fraction, %	64.4(5.1)
**ASA class**	
II	25(28)
III	47(53)
IV	16(18)
**Underlying aortic disease**	
Degenerative aneurysm	52 (59)
Post-dissection aneurysm	18 (20)
Penetrating ulcer	18 (20)
**Beginning of lesion**	
Z0	15 (17)
Z1	32 (36)
Z2	41 (47)
**Arch type**	
I	13 (14)
II	37 (41)
III	38 (42)
Maximum aortic diameter, mm	57.7(16.2)
Diameter of ascending aorta, mm	39.2 (4.8)
Maximum diameter ≥38 mm	53 (60)
Length of ascending aorta, mm	73.8(8.8)

CABG, coronary artery bypass grafting; eGFR, estimated glomerular filtration rate; PTCA, percutaneous transluminal coronary angioplasty.

### Procedure details

Technical success was achieved in all 88 patients (100%). Eighty patients (91%) received elective procedures, whereas 8 patients received emergent procedures. All procedures were performed under general anaesthesia with no preventive CSF drainage performed in any cases. Controlled hypotension during the deployment of the ascending aortic stent-graft was induced using vasodilators in 74 patients (85%) and rapid ventricular pacing in 14 patients (15%). After angiographic evaluation, 64 patients (73%) underwent simultaneous (70%) or staged (2%) LCCA-to-LSA bypass. The details of artery access are shown in *Table 2*.

**Table 2:** Procedural details (*n* = 88)

Variable	No. (%), mean(s.d.) or median (range; i.q.r.)
Technical success	88 (100.0)
Emergency case	8 (9)
General anaesthesia	88 (100)
CSF drainage	0 (0)
**Hypotension during deployment**	
Pharmacological	74 (85)
Rapid-pacing	14 (15)
**LCCA–LSA bypass**	
Simultaneous	62 (70)
Staged	2 (2)
None	24 (27)
**Access for IA extension**	
Right brachial artery	44 (50)
Right axillary artery	18 (20)
Right carotid artery	26 (30)
**LCCA access**	
Percutaneous	7 (8)
Open	81 (92)
Total operating room time, min	337.6(117.8)
Total fluoroscopy time, min	51.3(31.6)
Estimated blood loss, ml	200 (range, 50–1000; i.q.r., 150–400)
Volume of contrast used, ml	216.0(68.0)
Diameter of ascending aortic stent-graft, mm	40.6(4.3)
**Length of ascending aortic stent-graft**	
50 mm	66 (75)
60 mm	22 (25)
Distal diameter of aortic arch stent-graft, mm	30.9(3.8)
**IA bridging stent**	
>1 bridging stent	4 (5)
Tapered bridging stent	83 (94)
Distal diameter, mm	16 (range, 12–24; i.q.r., 15.5–18)
Length, mm	100 (range, 80–130; i.q.r., 100–120)
**LCCA bridging stent**	
>1 bridging stent	3 (3)
Tapered bridging stent	78 (89)
Distal diameter, mm	9 (8–12)
Length, mm	120 (range, 100–140; i.q.r., 120–120)
**LSA management**	
Ligation	10 (11)
Embolization	78 (89)

IA, innominate artery; LCCA, left common carotid artery; LSA, left subclavian artery.

The mean operating room time and fluoroscopy time were 337.6 ± 117.8 and 51.3 ± 31.6 min respectively. The mean contrast volume used was 216.0 ± 68.0 ml. The mean diameter of the ascending aortic stent-graft and distal segment of the aortic arch stent-graft were 40.6 ± 4.3 and 30.9 ± 3.8 mm respectively. Bridging stent information for the supra-aortic branches is summarized in *Table 2*.

### Early postoperative outcomes

Three patients died within 30 days post surgery: one owing to major stroke, one to respiratory failure, and one following sudden death after discharge, with suspected aortic-related death. None of the patients had conversion to open surgery or retrograde type A aortic dissection. Major adverse events occurred in 16 patients (18%) and included respiratory failure in 1 patient (1%); any stroke in 8 patients, including 1 fatal stroke event (9%); acute kidney injury in 1 patient (1%); and acute coronary syndrome and estimated blood loss >1 l in 3 patients each (3%) (*Table 3*). None of the patients developed spinal cord ischaemia or new-onset dialysis.

**Table 3:** Thirty-day outcomes

Outcome	No. (%) or median (range; i.q.r.)
All-cause death	3 (3)
Possible aortic-related death	1 (1)
Conversion to open surgery	0 (0)
Retrograde type A dissection	0 (0)
Acute coronary syndrome	3 (3)
Respiratory failure	1 (1)
Estimated blood loss >1 l	3 (3)
**Stroke**	
Major stroke	5 (6)
Minor stroke	3 (3)
Spinal cord ischaemia	0 (0)
Acute kidney injury	1 (1)
New-onset dialysis	0 (0)
**Iliofemoral access complications**	
Haematoma	4 (5)
Dissection	3 (3)
Pseudoaneurysm	3 (3)
Cervical haematoma	8 (9)
**Endoleak**	
I	0 (0)
II	7 (8)
III	0 (0)
IV	0 (0)
**Early reintervention**	
Cervical haematoma	5 (6)
Length of ICU stay, days	1 (range, 0–33; i.q.r., 0–3)
Length of hospital stay, days	18 (range, 7–45; i.q.r., 12–23)

ICU, intensive care unit.

The median hospital length of stay was 18 (range, 7–45; i.q.r., 12–23) days and the median length of stay in the intensive care unit was 1 (range, 0–33; i.q.r., 0–3) days. A total of 10 iliofemoral access complications occurred: 4 iliac artery haematomas, 3 iliac artery dissections, and 3 femoral artery pseudoaneurysms. Three patients with iliac artery haematoma and one with iliac artery dissection underwent concurrent endovascular repair during the index procedure. Cervical haematomas occurred in eight patients, five of whom required early reintervention. Postoperative CTA at 1 month identified seven type II endoleaks, none of which underwent early reintervention.

### Follow-up

The mean follow-up length was 28.6 ± 11.7 months. All patients underwent surveillance CTA except the three patients who died in the early postoperative period. Throughout the follow-up period, no stent-graft migration or structural failure was observed in any patient, and all bridging covered stents remained patent.

**Fig. 2:**
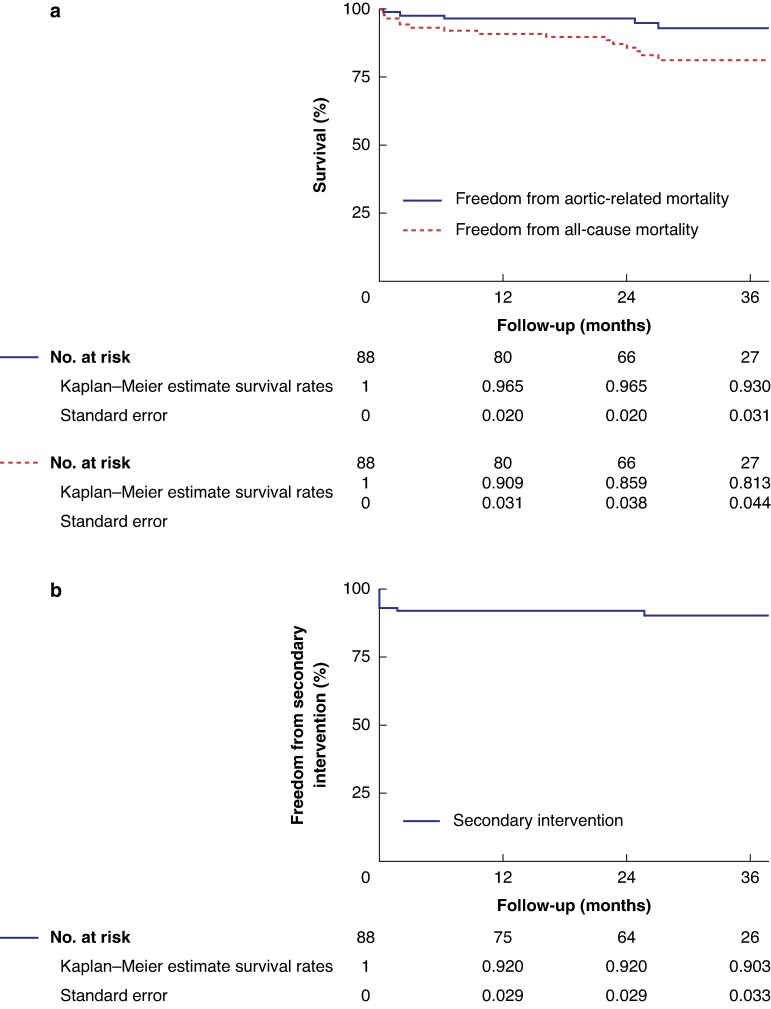
Kaplan–Meier analysis of survival (a) and freedom from secondary intervention (b)

During the follow-up period, 12 patients died. Eight deaths were non–aortic-related, whereas four were deemed aortic-related. Among the aortic-related deaths: two were from severe infection secondary to aortoesophageal fistula; one occurred after sudden abdominal pain, with a suggested ruptured abdominal aortic aneurysm; and one occurred after acute onset of chest and back pain, presumed to be cardiac- or aortic-related. Overall survival was 91% ± 3%, 86% ± 4%, and 81% ± 4% at 12, 24, and 36 months respectively. Freedom from aortic-related death was 97% ± 2%, 97% ± 2%, and 93% ± 3% at 12, 24, and 36 months respectively (*Fig. 2a*).

Three patients required a secondary intervention during follow-up. One patient underwent patch angioplasty for a femoral artery pseudoaneurysm. One patient underwent endovascular repair of a thoracic aortic aneurysm as part of a planned staged procedure. One patient underwent bronchial artery embolization for haemoptysis. Freedom from reintervention was 92% ± 3%, 92% ± 3%, and 90% ± 3% at 12, 24, and 36 months respectively (*Fig. 2b*).

A total of 10 patients developed endoleaks, all classified as type II and presumed to originate from the LSA or bronchial arteries, with no secondary intervention required. Among the seven patients with type II endoleaks detected on the 30-day postoperative CTA, six exhibited persistent minor endoleaks without enlargement of the aneurysm and one patient had a minor endoleak that spontaneously resolved within 12 months of the procedure. Of the three patients without endoleak on the 30-day CTA, one had an endoleak that was first identified at 6 months after the procedure (without aneurysm enlargement); one at 12 months (with sac regression); and one at 24 months (with sac regression).

### Risk factor analysis and comparative analysis between high-volume and low-volume centres

Significant differences were observed between high-volume and low-volume centres in terms of operative time and contrast volume (*P* < 0.05). Although no statistically significant differences were found in complication rates, a trend towards higher stroke incidence was noted in low-volume centres (*P* = 0.067). The other results are summarized in *Table S3*.

The relationship between ascending aortic diameter and clinical outcomes is presented in *Table S4*. Univariate logistic regression analysis showed no significant association between an ascending aortic diameter ≥38 mm and early death and stroke. This non-significant association persisted after adjustment for potential confounding variables.

## Discussion

This study features the largest Asian cohort to date of patients with aortic arch aneurysm treated with an off-the-shelf inner-branched endovascular stent-graft. Our findings demonstrate the safety and effectiveness of endovascular aortic arch repair using the WeFlow-Arch (Endonom Medtech, Hangzhou, China) modular inner branched stent-graft in selected patients. Technical success was achieved in all patients, with low perioperative death (3%) and an acceptable stoke rate (9%). The early outcomes of this study demonstrate high technical success and low perioperative death, which are encouraging in comparison with the data for open surgical repair^1–5^.

Open surgical repair remains the gold standard for treating aortic arch pathology. In the recent expert consensus of the European Association for Cardio-Thoracic Surgery and the European Society for Vascular Surgery, endovascular aortic arch repair was recommended only for patients unfit for open surgery and with a suitable anatomy^14^. Our study included patients referred for endovascular repair after being deemed ineligible for open surgery by cardiac surgeons, as well as those who declined open surgery. Consequently, the study population encompassed individuals physically suitable for open procedures. We found no significant differences in early death and stroke between patients with ASA class II and class III/IV (8% *versus* 13%, *P* = 0.718). Long-term follow-up will provide evidence of the outcomes of endovascular aortic arch repair in patients eligible for open repair. If endovascular aortic arch repair is considered safe and effective during longer-term follow-up in patients not at high surgical risk, a consensus meeting with the participating surgeons, regulatory authorities, patients, and statisticians should be convened to discuss the feasibility of designing a large-scale RCT comparing endovascular *versus* open aortic arch repair.

Stroke remains a primary concern following endovascular aortic arch repair. In the COOK A-branch multicentre trial (which enrolled individuals with aortic aneurysms and chronic dissections), the perioperative stroke rate was 11%—comparable to the 9% observed in our study^20^. This similarity likely reflects both cohorts’ predominance of patients with aneurysms (72% and 80% respectively), who carry a higher stroke risk than patients with dissection. Conversely, an A-branch trial exclusively enrolling chronic dissections reported a 30-day stroke rate of only 3%^21^. This discrepancy may be attributable to lower atherosclerotic burden in dissection patients reducing stroke susceptibility and participation exclusively by high-volume centres (≥5 A-branch implantation experiences). Notably, the RELAY multicentre study (80% patients with aneurysms) demonstrated stroke rates as high as 26%^9^. Collectively, these findings highlight the critical need for refined patient selection and enhanced perioperative stroke prophylaxis strategies in future research.

Single-branched stent-grafts combined with supra-aortic branch bypass represent a common treatment strategy for aortic arch pathologies. The Nexus aortic arch stent-graft is another branched platform, characterized by its unique design featuring a main body with a single branch. It is deployed using a through-and-through wire from the femoral artery to the right brachial artery. An international multicentre study involving 28 patients reported a 30-day mortality rate of 7% and a 30-day stroke rate of 4%^22^. A similar approach involves using the GORE TBE device for brachiocephalic trunk reconstruction combined with supra-aortic bypass to address aortic arch lesions. Although the TBE was originally designed for LSA revascularization, its branch diameter of up to 20 mm and the use of a proximal cuff allow it to be adapted for the treatment of aortic arch pathologies^12^.

Compared with the unibody arch stent-graft, the modular inner branched stent-graft system has some potential advantages. First, with the split main body modular design, this stent-graft system accommodates more anatomic variations of the aortic arch, allowing it to be used off-the-shelf in both elective and urgent cases. Second, the step-by-step assembly procedures ensure persistent blood supply to the supra-aortic branch vessels, which may help reduce the risk of cerebrovascular events during surgery. A potential drawback may be the greater number of procedural steps involved.

Previous studies on endovascular aortic arch repair reported higher early mortality and stroke rates in patients with ascending aortic diameters ≥38 mm^19^. In this cohort, the mean ascending aortic diameter was 39.2 ± 5.1 mm, with 53 patients (60%) exceeding the 38 mm threshold. Multivariable regression analysis revealed no significant association between ascending aortic diameter (continuous variable) or diameter ≥38 mm (categorical variable) and early death and stroke (*P* > 0.05). This diverges from the prior literature and may be attributable to smaller sample sizes (*n* = 38) and univariate comparisons in previous reports *versus* adjusted logistic regression analysis of our larger cohort (*n* = 88). These results underscore the need for further large-scale investigations to clarify this relationship.

During the follow-up of this study, endoleaks were not rare (*n* = 10, 11%). Eight endoleaks were presumed to originate from the LSA and two from bronchial arteries. In this cohort, proximal LSA management was achieved with coil embolization in 78 cases, whereas only 10 cases involved surgical ligation. This represents a notably lower ligation rate compared with those in previous reports (11% *versus* 68%)^10^. The predominance of coil embolization may partially explain the endoleak observations: although coils markedly reduce flow, the potential for type II endoleaks persists. Fortunately, none of the 10 affected patients exhibited aneurysm enlargement during follow-up. Sac regression occurred in two patients, and spontaneous endoleak resolution was observed in one patient. Surgical ligation of the LSA necessitates deeper dissection of supra-aortic structures, potentially increasing procedural risk. The remaining seven patients with endoleaks require continued surveillance to provide evidence for proper intraoperative LSA management strategy for subsequent cases.

This study suggests the existence of a learning curve for this procedure, with significant differences between high-volume and low-volume centres observed in operating room time and contrast volume. Perioperative outcomes demonstrated no statistically significant disparities, although a trend towards divergence of stroke rate between the high-volume and low-volume groups was observed. This indicates that future initiatives should implement standardized training programmes, including supervised operating room observation and simulator-based practice, to accelerate the transition to proficiency.

Compared with previous studies, this study features a larger sample size and longer follow-up duration and includes centres performing endovascular aortic arch repair with off-the-shelf devices for the first time. Nevertheless, several limitations warrant consideration. Although this was a prospective study, the absence of a control group precludes direct comparative analysis between endovascular and conventional open surgical repair. Furthermore, the study enrolled a highly selected patient population from a single ethnic group, predominantly comprising individuals at high surgical risk, which limits the generalizability of our findings to the broader population.

Endovascular aortic arch repair using modular branched stent-grafts shows encouraging safety and efficacy, with promising short-term and midterm outcomes. This approach represents a reasonable alternative treatment for patients at high surgical risk. Long-term follow-up data and comparative studies directly benchmarked against open surgical repair are needed to support broader application of the procedure to general populations.

## Supplementary Material

znaf279_Supplementary_Data

## Data Availability

Because of the sensitive nature of the data collected for this study, requests to access the data set from qualified researchers trained in human subject confidentiality protocols may be sent to guoweiplagh@sina.com.
