# Supplementary material for: Outcomes of endovascular aortic arch repair with an off-the-shelf modular inner branched stent-graft: an IDEAL 2a prospective multicentre trial
Source: Br J Surg. 2026 Jan 9;113(1):znaf279. doi: 10.1093/bjs/znaf279 (PMC12785883; doi:10.1093/bjs/znaf279)
Supplement: znaf279_Supplementary_Data [file znaf279_supplementary_data.docx]

**Outcomes of endovascular aortic arch repair with an off-the-shelf modular inner branched stent-graft: an IDEAL 2a prospective multicentre trial**

Wei Guo^1,2,†^, Dan Rong^1,^^†^, Hongkun Zhang^3^, Leiyang Zhang^4^, Hui Zhuang^5^, Hua Peng^5^, Xuejun Wu^6^, Kunmei Gong^7^, Wei Wang^8^, Zhen Li^9^, Weiguo Fu^10^, Xiaoming Zhang^11^, Mingjin Guo^12^, Guangqi Chang^13^, Xiangchen Dai^14^, Jian Zuo^15^, Yingqiang Guo^16^, Bing Chen^17^, Lei Zhang^18^, Taoran Zhang^2^, Hongpeng Zhang^1^

^1^Department of Vascular and Endovascular Surgery, Chinese PLA General Hospital, Beijing, China

^2^Nankai University School of Medicine, Tianjin, China

^3^Department of Vascular Surgery, The First Affiliated Hospital of Zhejiang University School of Medicine, Hangzhou, China

^4^Department of Thoracic and Cardiovascular Surgery, Nanjing First Hospital, Nanjing, China

^5^Department of Vascular Surgery, Xiamen Cardiovascular Hospital Xiamen University, Xiamen China

^6^Department of Vascular Surgery, Shandong Provincial Hospital, Jinan, China

^7^Department of Vascular Surgery, The First People’s Hospital of Yunnan Province, Kunming, China

^8^Department of Vascular Surgery, Xiangya Hospital of Central South University, Changsha, China

^9^Department of Endovascular Surgery, The First Affiliated Hospital of Zhengzhou University, Zhengzhou, China

^10^Department of Vascular Surgery, Fudan University Affiliated Zhongshan Hospital, Shanghai, China

^11^Department of Vascular Surgery, Peking University People’s Hospital, Beijing, China

^12^Department of Vascular Surgery, The Affiliated Hospital of Qingdao University, Qingdao, China

^13^Department of Vascular Surgery, The First Affiliated Hospital of Sun Yat-sen University, Guangzhou, China

^14^Department of Vascular Surgery, Tianjin Medical University General Hospital, Tianjin, China

^15^Department of Cardiovascular Surgery, The First Affiliated Hospital of Air Force Medical University, Xi’an, China

^16^Department of Cardiovascular Surgery, West China Hospital, Chengdu, China

^17^Department of Vascular Surgery, The Second Affiliated Hospital of Zhejiang University School of Medicine, Hangzhou, China

^18^Department of Vascular Surgery, The First Hospital of Hebei Medical University, Shijiangzhuang, China

† The first two authors contributed equally to this work.

**Corresponding author:** Wei Guo, Department of Vascular and Endovascular Surgery, Chinese PLA General Hospital, 28 Fuxing Road, Beijing 100853, China (E-mail: [guoweiplagh@sina.com)](mailto:guoweiplagh@sina.com))

**ORCID ID**：0000-0001-6212-8390

**Supplementary Materials - Index**

| **Supplementary Methods** |  |
| --- | --- |
| NA | *NA* |
| NA | *NA* |
| **Supplementary Results** |  |
| NA | *NA* |
| NA | *NA* |
| **Supplementary Appendixes** |  |
| NA | *NA* |
| NA | *NA* |
| **Supplementary Figures and Tables** |  |
| Figure S1 | *page 3* |
| Figure S2 | *page 4* |
| Figure S3 | *page 5* |
| Table S1 | *page 6-7* |
| Table S2 | *page 8* |
| Table S3 | *Page9* |
| Table S4 | *page 9-10* |
| **References** |  |
| NA | *NA* |

**Supplementary Figures and Tables**

**
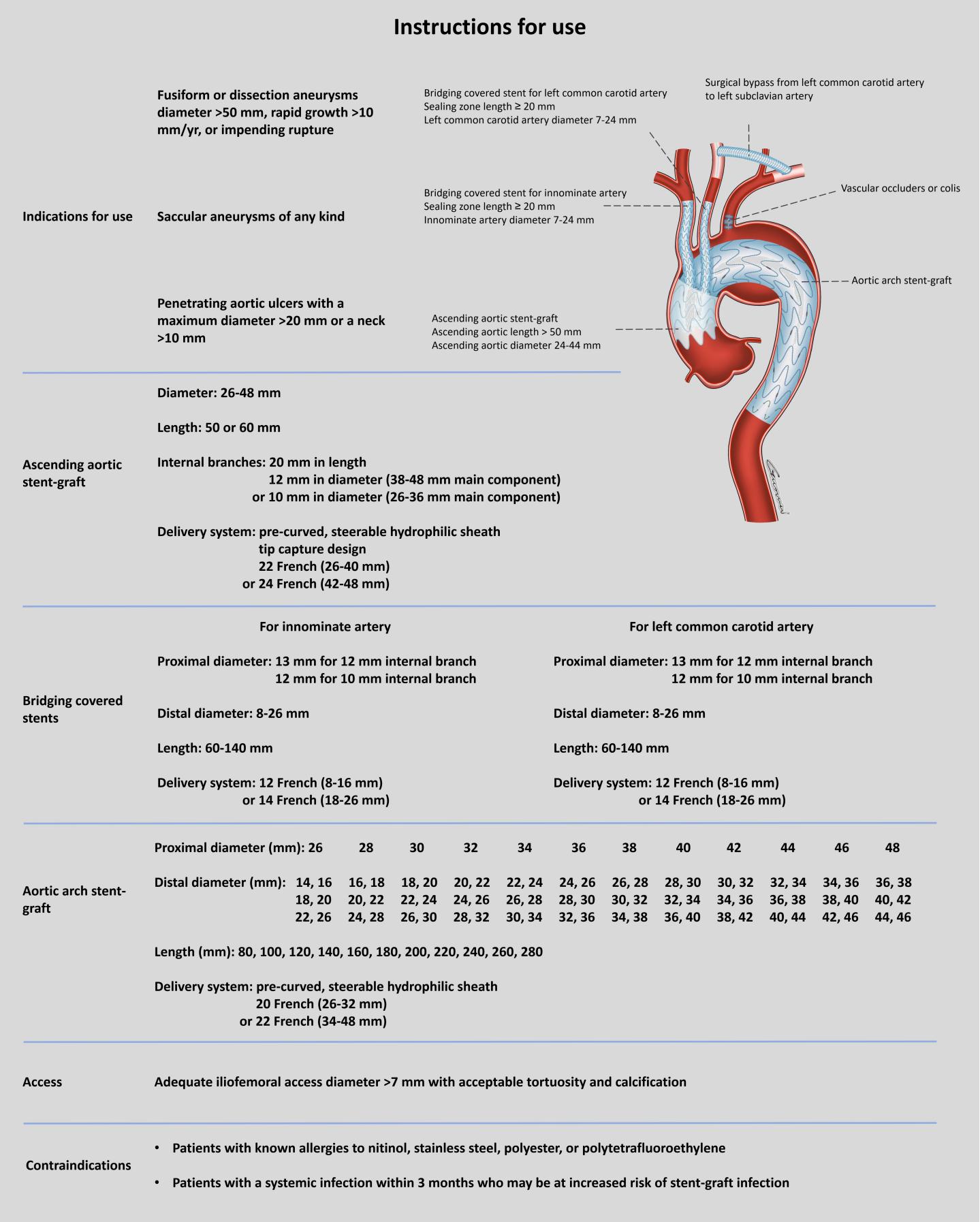
**

Fig. S1. Instructions for use of the WeFlow-Arch modular branched stent-graft system.

**
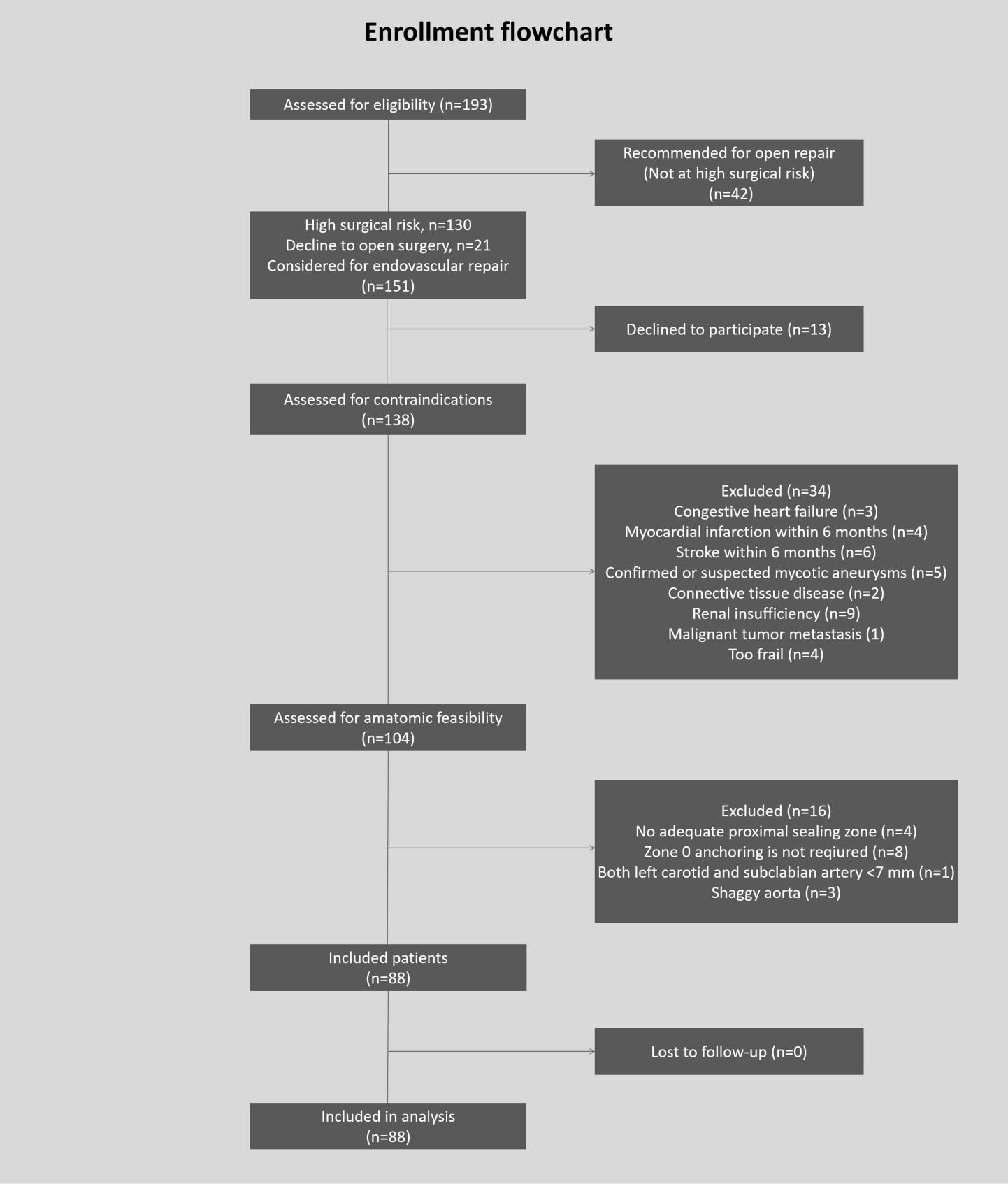
**

Fig. S2. Flowchart of patients enrollment.

**
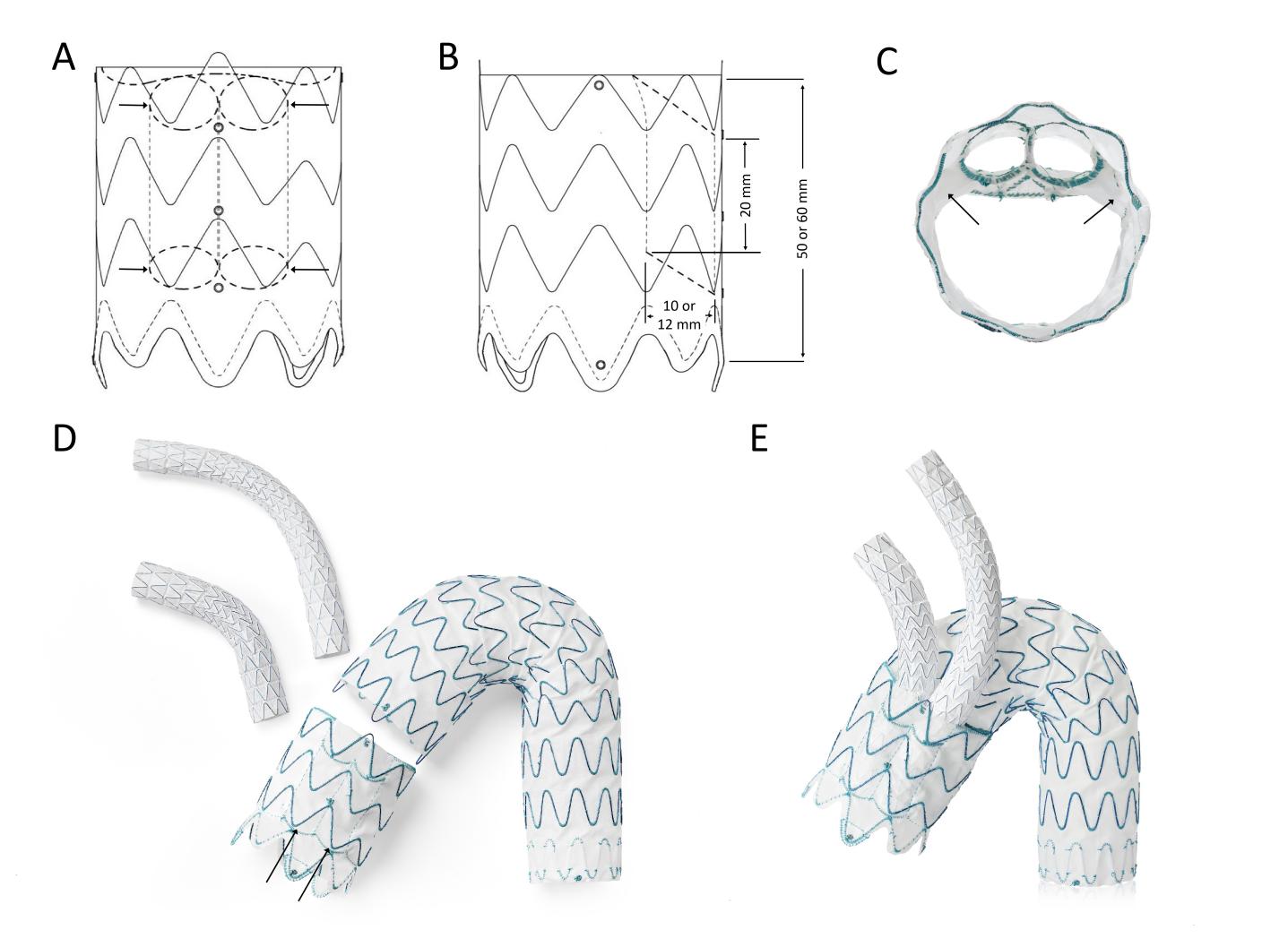
**

Fig. S3. Design schematic and physical photograph of the stent-graft. A, Anterior view of the ascending aortic stent-graft, four black arrow indicate the ring markers on both ends of inner branches. B, Lateral view of the ascending aortic stent-graft. C, Internal view of the ascending aortic stent-graft, black arrows indicate that the gutter between main tunnel and inner branches were sealed by stitched membrane. D, Separated components of the modular inner branch stent-graft system, black arrows indicate two inner branches at 8:00-o’clock and 10:00-o’clock position, respectively. E, Assembled components of the modular inner branch stent-graft system.

Table S1. IDEAL Checklists for IDEAL Stages 2a.

| **STAGE 2a** | **Item** | **Checklist Item for key IDEAL items** |  |
| --- | --- | --- | --- |
| Title and Abstract | 1a | Identification as a prospective case series of a novel technique in the title, including the IDEAL stage in the title or abstract. | Yes |
|  | 1b | Provide a structured summary of background, methods, results, and conclusions. | Yes. Page 4-5. |
| **Introduction** |  |  |  |
| Background and Objectives | 2a | Review of existing scientific literature, including reference to IDEAL Stage 1 and 2a reports in previous publications, if applicable. | Yes. Page 6. |
|  | 2b | Specific objectives stated, including refining the technique and progressing toward stability. | Yes. Page 7. |
| **Methods** |  |  |  |
| Design | 3 | Description of study design (e.g. sequentially reported prospective case series). | Yes. Page 7. |
| Participants | 4a | Detailed account of patient inclusion and exclusion criteria. | Yes. Page 8-9. |
|  | 4b | Informed consent process described, including explanation of risks and acknowledgement of level of experience with technique/device. | Yes. Page 7. |
|  | 4c | Setting, location, and timeframe of recruitment and follow-up, including when and where the data were collected, as well as hospital characteristics and appropriate details regarding the operator/team (e.g. prior experience with novel technique). | Yes. Page 8 and Table S1. |
| Intervention | 5a | Clear and detailed description of (or reference to) planned technique, including necessary pre-operative and post-operative care. | Yes. Page 10-12. |
|  | 5b | Patient safety monitoring methods and safeguards. | Yes. Page 12. |
| Outcomes | 6 | Description of outcome measure(s) selected and how they will be assessed, including patient reported outcome measures, when appropriate, utilising those measures that are standardised and validated, when available and applicable. When these are not available, provide rationale for the outcome measure(s) used. | Yes. Page 12-13. |
| **Results** |  |  |  |
| Baseline data | 7 | Patient baseline demographic and clinical characteristics, including how many patients were assessed for treatment and a description of which patients were included, excluded, or refused, and why (to be displayed in a flow diagram format, when appropriate). | Yes. Page 14. |
| Intervention | 8 | Transparent reporting of all cases in the sequence they were performed, clearly indicating when and why modifications to the technique took place, including visual aids of the technique and modifications (e.g. photographs, videos, etc) when available. | Yes. Page 14. |
| Outcomes | 9 | Technical, clinical and patient-reported outcomes described for each patient, with all available outcome data incorporated into a comprehensive table or graph, whenever possible, to allow for the relationship to be clearly visualized between technique modifications and outcomes. | Yes. Page 15-17. |
| Harms | 10 | Transparent account of all harms or unintended effects reported for each patient. | Yes. Page 15-16. |
| **Discussion** |  |  |  |
| Interpretation | 11 | Analysis of technique development, including consistency of results and a balanced discussion of benefits and harms. | Yes. Page 17,19. |
| Limitations | 12 | Study limitations, addressing sources of potential bias. | Yes. Page 21. |
| Stage End-Points | 13a | Have the technique and outcomes reached stability in the hands of the current team (e.g. there is no intent to make further major modifications to the technique, and patient outcomes are stable)? Include an explanation of how you determined stability. | Yes. Page 21. |
|  | 13b | Discussion of whether the technique is ready for evaluation in a prospective, multi-centre IDEAL Stage 2b study, and identification of indications for the technique. | Yes. Page 18. |
| Conclusions | 14 | Conclusions and relevance, including plans to progress to future IDEAL stages, if applicable. | Yes. Page 21. |
| **Other Information** |  |  |  |
| Protocol | 15e | Please quote reference or DOI if a protocol was written in advance and made available. If a protocol was not made available, consider including as a supplement if the journal allows. | Yes. Page 7. |
| Ethics | 15f | Reference to ethical approvals obtained, and independent oversight, if applicable. | Yes. Page 7. |
| Funding | 15g | Sources of funding and support, role of funders, and other conflicts of interest. | Yes. Page 3. |
| Regulatory Approvals | 16 | Regulatory approvals being sought or obtained (e.g. CE Marking, FDA approval, etc) including the date of approval, if applicable. | Yes. Page 7. |

Table S2 Participant centers

| Institution | Patients included |
| --- | --- |
| Chinese PLA Gegeral Hospital | 16 |
| The First Affiliated Hospital of Zhejiang University School of Medicine | 12 |
| Nanjing First Hospital | 12 |
| Xiamen Cardiovascular Hospital Xiamen University | 6 |
| Shandong Provincial Hospital | 5 |
| The First People’s Hospital of Yunnan Province | 5 |
| Xiangya Hospital of Central South University | 5 |
| The First Affiliated Hospital of Zhengzhou University | 5 |
| Fudan University Affiliated Zhongshan Hospital | 4 |
| Peking University People’s Hospital | 4 |
| The Affiliated Hospital of Qingdao University | 4 |
| The First Affiliated Hospital of Sun Yat-sen University | 3 |
| The First Affiliated Hospital of Air Force Medical University | 2 |
| Tianjin Medical University General Hospital | 2 |
| The Second Affiliated Hospital of Zhejiang University School of Medicine | 1 |
| West China Hospital | 1 |
| The First Hospital of Hebei Medical University | 1 |
| Total | 88 |

Table S3. High volume vs low volume group

|  | High volume group (n=40) | Low volume group (n=48) | P value |
| --- | --- | --- | --- |
| Emergency case | 6 (15) | 2 (4) | 0.134 |
| Operating room time, min | 282.4 ± 77.9 | 383.5 ± 126.2 | < 0.001 |
| Volume of contrast used, mL | 197.2 ± 67.4 | 230.2 ± 65.1 | 0.023 |
| 30-day mortality | 1 (3) | 2 (4) | 1 |
| Stroke | 1 (3) | 7 (12) | 0.067 |
| Early mortality and any stroke | 2 (5) | 8 (17) | 0.104 |
| Iliofemoral access complications | 3 (8) | 7 (15) | 0.336 |
| Cervical haematoma | 3 (8) | 5 (10) | 0.723 |
| Re-intervention | 1 (3) | 5 (10) | 0.214 |

Table S4. Multivariable logistic regression models evaluating the association between ascending aortic diameter and early mortality and stroke.

| Variable | Total No. | No. of event | OR (95%CI) | | P value | |
| --- | --- | --- | --- | --- | --- | --- |
|  |  |  | Crude | Adjusted* | Crude | Adjusted* |
| Ascending aortic diameter, 1 mm | 88 | 10 (11) | 1.03 (0.89~1.19) | 1.06 (0.90~1.24) | 0.673 | 0.497 |
| Ascending aortic diameter <38 mm | 35 | 4 (11) | Ref | Ref |  |  |
| Ascending aortic diameter≥38 mm | 53 | 6 (11) | 0.99 (0.26~3.79) | 1.05 (0.22~5.03) | 0.988 | 0.947 |
| *Adjusted for arch type, aortic diameter, beginning of lesion, history of cerebral artery. OR, odds ratio. | | | | | | |
